# Determining the role of SGLT2 inhibition with Empagliflozin in the development of diabetic retinopathy

**DOI:** 10.1042/BSR20212209

**Published:** 2022-03-02

**Authors:** Jennifer Matthews, Lakshini Herat, Jennifer Rooney, Elizabeth Rakoczy, Markus Schlaich, Vance B. Matthews

**Affiliations:** 1 Dobney Hypertension Centre, School of Biomedical Sciences – Royal Perth Hospital Unit, University of Western Australia, Crawley, WA 6009, Australia; 2 Department of Molecular Ophthalmology, University of Western Australia, Crawley, WA 6009, Australia; 3 Dobney Hypertension Centre, School of Medicine – Royal Perth Hospital Unit, University of Western Australia, Crawley, WA 6009, Australia; 4 Department of Cardiology and Department of Nephrology, Royal Perth Hospital, Perth WA 6000, Australia

**Keywords:** Diabetes, Empagliflozin, Opthamology, SGLT2

## Abstract

Diabetes mellitus is a chronic metabolic disease that occurs when the pancreas is not producing enough insulin or when the insulin that it does produce is not able to be used effectively in the body. This results in hyperglycemia and if the blood sugars are not controlled, then it can lead to serious damage of various body systems, especially the nerves and the blood vessels. Uncontrolled diabetes is a major cause of kidney failure, heart attacks, stroke and amputation. One of the most devastating complications for patients is diabetic retinopathy (DR) which represents the leading cause of preventable vision loss in people between 20 and 65 years of age. Sodium glucose transporter 2 (SGLT2) inhibitors have been shown to reduce the risk for cardiovascular and renal events, however literature highlighting their potential role to prevent DR is limited. We therefore used a relevant mouse model (Akimba) to explore the effects of the SGLT2 inhibitor, Empagliflozin (EMPA), on the development of diabetic retinal changes. Here we show that when given in the early stages of type 1 diabetes (T1D), EMPA reduced the weight loss usually associated with T1D, decreased diabetes-associated polydipsia, lowered fasting blood glucose levels, decreased kidney-to-body weight ratios and, most importantly in the current context, substantially reduced retinal abnormalities associated with DR. We show that EMPA reduces vascular leakage indicated by lower albumin staining in the vitreous humor and diminishes expression of the pathogenic factor VEGF in the retina. Additionally, EMPA significantly alters the retinal genetic signature. Our findings suggest that SGLT2 inhibition may be a useful therapeutic approach to prevent the development of DR and its severity if given early in the disease process.

## Introduction

In 2017, diabetes accounted for 9.9% of all-cause mortality worldwide (approximately 5 million deaths) [1]. Diabetes is a major cause of kidney failure, heart attacks, stroke, amputation and diabetic retinopathy (DR) [2]. DR occurs as a result of long-term accumulated damage to the small blood vessels in the retina and accounts for 2.6% of global blindness [3]. On a global scale, the number of people with DR is estimated to grow from 126.6 million in 2011 to 191 million in 2030, with the number of vision-threatening cases increasing from 37.3 million in 2010 to 56.3 million in 2030 [2]. Clinically, DR is divided into two stages: non-proliferative DR (NPDR) and proliferative DR (PDR). DR is both a vascular and neurodegenerative disease of the retina [4]. The NDPR is characterized by capillary occlusion, microaneurysms, intraretinal microvascular abnormalities (IRMAs) and hemorrhages, while PDR is characterized by the hallmark feature of pathologic retinal neovascularization [5]. These proliferative changes are usually accompanied by unavoidable vision loss.

At present, the three major therapeutic strategies effective in reducing DR-induced vision impairment include anti-vascular endothelial growth factor (VEGF) injections, panretinal laser photocoagulation and vitrectomy [6]. As these therapies are not always effective and associated with various complications, additional effective therapies are warranted and novel therapeutic targets are sought for treatment and prevention of DR [7]. Anti-VEGF treatment has been found to be very effective in the rapid reduction in retinal neovascularization in patients with PDR and reduces macular edema. However, it requires frequent intravitreal injections for several years and is therefore costly [6]. Furthermore, anti-VEGF therapy has potential complications and does not reverse the underlying pathology of DR [8]. Current DR treatment options are largely focused on the end stage of the disease and does not address the early and possibly reversible microvascular changes leading to DR. Therefore, therapeutic approaches that can be used in the early stages of the disease process, to prevent or slow down the progression of DR, would be most likely to result in a reduction in the devastating consequences of DR [8]. As diabetes and its consequences are predominantly a result of excessive glucose levels in the bloodstream, a therapy that reduces glucose levels and improves insulin sensitivity while also affecting the pathophysiological pathways implicated in DR would be most useful. Sodium glucose transporter 2 (SGLT2) inhibitors clearly fulfil the first two criteria [9], however, whether they can also prevent the development of DR has not yet been thoroughly investigated. We have shown that SGLT2 inhibitors mediate their cardiovascular and renoprotective effects at least in part by sympathoinhibition [10], a pathway that may also be relevant for DR. It is well known that the sympathetic nervous system can influence endothelial function and ultimately ocular blood circulation. It has been shown that ocular sympathetic nerves are critical for the generation and maintenance of immune privilege in the eye through the facilitation of local transforming growth factor-β production [11]. The transforming growth factor-β is a vital ligand involved in the modulation of cell migration and proliferation, cell death and protein synthesis during development, tissue repair and other physiological or pathological processes in the eye [11]. Additionally, increased blood pressure via increased blood flow may damage the retinal capillary endothelial cells in eyes of people with diabetes. Therefore, it is plausible that SGLT2-mediated sympathoinhibition may be beneficial for DR. Future studies in the Akimba mouse model are required.

In the present study, we examine the influence that the SGLT2 inhibitor, Empagliflozin (EMPA), has on the prevention and development of retinopathy in a mouse model of early DR.

## Materials and methods

### Mice and genotyping

As a model system, we utilized the Akimba mouse (Ins2^Akita^VEGF^+/−^), a novel transgenic model of DR that was generated by crossing the diabetic Akita (Ins2Akita) which develops hyperglycemia with Kimba (trVEGF029) mice. The Kimba mouse has transient photoreceptor-specific overexpression of human vascular endothelial growth factor 165 (VEGF165) and hence depicts changes associated with DR without the hyperglycemic background. The Akimba mouse (Ins2AkitaVEGF^+/−^) is a cross between the diabetic Akita and the Kimba model, and develops advance DR features due to the interplay between high blood glucose levels and overexpression of VEGF [4]. The Akimba mouse model displays hallmark retinal microvascular abnormalities representative of human DR including microaneurysms, tortuous vessels, venous beading, vasodilation, capillary non-perfusion, increased retinal vascular leakage, edema and neovascularization [4,12,13]; this model is a highly relevant model for the study of retinal vascular disease. DNA was isolated from tail clips by using the Wizard Genomic DNA Purification Kit (Promega, Madison, WI). Genotyping of Kimba mice was carried out as previously described [14,15]. Akita mice were genotyped for the insulin 2 (*Ins2*) gene as described [16]. Akimba mice were genotyped by using protocols for both Kimba and Akita mice.

### Animal experimentation

Animal experimentation was carried out at the Harry Perkins Institute for Medical Research animal holding facility (Perth, WA). Animal ethics was approved by the Harry Perkins Institute for Medical Research Animal Ethics Committee (AE141/2019) and all procedures were performed in accordance with the Association for Research in Vision and Ophthalmology statement for use of animals in ophthalmic and vision research. Male Kimba and Akimba mice were bred and obtained from the Animal Resources Centre (Perth, WA, Australia) at 2–5 weeks of age. Mice were housed under a 12-h light/dark cycle, at 21 ± 2°C and were given a standard chow diet (Specialty Feeds, Glen Forrest, WA, Australia) with free access to food and water. Wet mash was replenished three times per week. Following a 7-day acclimatization period, the SGLT2 inhibitor EMPA (Ark Pharma Scientific Limited, China; 25 mg/kg/day) or vehicle (dimethylsulfoxide) was administered to the mice weekly via their drinking water for 8 weeks. Urine glucose levels were measured (Keto-Diastix; Bayer, Leverkusen, Germany) before treatment and 1 week post-treatment to establish the diabetic status of Akimba mice and the development of glucosuria induced by EMPA. Mice were weighed weekly. Fasting blood glucose levels were measured at the end of the experiment using the Accu-Chek Performa blood glucose monitoring system (Roche Diagnostics, North Ryde, Australia). Only male mice were used in the present study as disease progression is known to be slower and less uniform in female mice [17].

### Tissue and serum sample collection

After 8 weeks of their respective treatments, the mice were fasted for 5 h and had access to treatment water. Mice were deeply anesthetized using isoflurane inhalation, underwent cardiac puncture to obtain blood and were killed by cervical dislocation. Blood samples were centrifuged, serum collected and stored at −80°C. Kidneys were harvested, weighed and fixed in 10% buffered paraformaldehyde (PFA) for histology. The right eye was collected into 10% buffered PFA for histology while the left eye was fixed in ice-cold 10% buffered PFA for 2 h at 4°C in preparation for retinal vascular assessment.

### Gene expression assays

Firstly, the kidney medulla and cortex were dissected using springbow scissors while slightly frozen. RNA from murine eyes and kidneys was extracted using TRIzol reagent (Invitrogen, Thermo Fisher, Melbourne, Victoria, Australia) and cDNA synthesis was performed using the High Capacity RNA-to-cDNA kit (Applied Biosystems, Thermo Fisher, Melbourne, Victoria, Australia). Real-time PCR was performed to determine the mRNA abundance utilizing a Rotor-gene real-time PCR machine (Qiagen, Hilden, Germany) using pre-developed TaqMan probe (FAM-labeled) and primer sets for *Hprt* (Mm01545399_m1) and *Sglt2* (*Slc5a2*; Mm00453831_m1) (Applied Biosystems). Quantitation was conducted as previously described [18].

### Retinal isolation and whole mount preparation

Retinal flat-mounts were prepared with modification to a previously published method [19]. In brief, the fixed eyes were washed in ice cold 1× PBS for 2 min and retinas were dissected in 1–2 ml of ice cold 1× PBS under a dissection microscope. The fully dissected retinas were washed twice in 1× PBS prior to staining. Fixed retinas were permeabilized for 2 h in PBS-BSA-Triton-X (1× PBS, 1% BSA, 1% Triton-X) at room temperature followed by washing in Wash Buffer (1× PBS, 0.5% Triton-X) for five times. Retinas were incubated overnight in the dark at 4°C with biotinylated GSL-1B4 (1:100 in Wash Buffer) with specificity for α-galactosylated glycoprotein residues on vascular endothelial cells and macrophages (*Griffonia Simplicifolia* Lectin I (GSL I) isolectin B4; Catalog#: B-1205-.5, Biotinylated; Vector Laboratories, Burlingame, CA, U.S.A.). Retinas were washed five times in 1× PBS and secondary antibody Cy3-Streptavidin (1:500 in 1× PBS; GE Healthcare, Amersham, U.K.) was added and incubated for 5 h in the dark at room temperature. Stained retinas were washed five times in 1× PBS, mounted and coverslipped with VECTASHIELD HardSet Anti-fade Mounting Medium (Vector Laboratories, Burlingame, CA, U.S.A.).

### Retinal imaging and analysis

Retinal whole-mounts were imaged using the inverted fluorescent microscope Nikon Eclipse Ti (Nikon, Tokyo, Japan) with a digital camera CoolSNAP HQ2 (Photometrics, Tucson, AZ, U.S.A.) linked to a computer running the image analysis software ‘NIS-Elements Advanced Research’ (Nikon, Tokyo, Japan). Sequentially overlapping high-resolution images of the entire retinas were captured using the 4× objective. Images were merged to construct a montage of the retina. Montages were semi-quantified for vascular morphological abnormalities as previously published [4]. Grading of retinas were based on vascular changes such as regions of capillary dropout, vascular leakage, vessel tortuosity, monocytes/macrophages, microaneurysms, IRMA, exceptionally dense vascular budding and neovascular tufts. Representative areas of the retinal vascular lesions were captured in the z-plane in 2-μm steps. The z-stacks were compiled into a focused image representing all superficial, intermediate and deep capillary layers.

### SGLT2 immunohistochemistry

The detection of SGLT2 protein expression in eyes was conducted. Briefly, eyes were fixed in 10% buffered formalin for 24 h, followed by wax embedding. Paraffin sections (5 μm) were collected and mounted on slides. Eyes were washed in xylene (2 × 10 min) and then rehydrated in 100% ethanol (2 × 5 min), 95% ethanol (1 × 5 min), 70% ethanol (3 min) and then water (5 min). For antigen retrieval, slides were heated for 5 min in a pre-heated 1× EDTA buffer (pH 8.5; Sigma–Aldrich, Sydney, Australia). After washing twice in PBS/0.1% Tween for 5 min, tissue sections were outlined with a paraffin pen. Sections were blocked with 3% H_2_O_2_ for 10 min, washed twice with PBS/0.1% Tween for 5 min and blocked with 5% FBS in PBS/0.1% Tween for 1 h in a humidified chamber. Sections were then incubated overnight at 4°C in a humidified chamber with both rabbit anti-SGLT2 (1:200; Novus Biologicals, Colorado, U.S.A.) and mouse anti-SGLT2 antibodies (1:50; Santa Cruz Biotechnology, California, U.S.A.) in 5% FCS/PBS/0.1% Tween. Following overnight incubation, sections were washed three times with PBS/0.1% Tween for 5 min and incubated with anti-rabbit (1:100, Santa Cruz Biotechnology, Sydney, Australia) and anti-mouse (1:100, Santa Cruz Biotechnology, Sydney, Australia) secondary antibodies conjugated with HRP in PBS/0.1% Tween for 1 h. This was followed by incubation with diaminobenzidine (DAB). Slides were counterstained with Hematoxylin, dehydrated and mounted with DPX (Sigma–Aldrich, Sydney, Australia).

### Albumin and VEGF immunohistochemistry

The detection of albumin and VEGF protein in the Akimba mice was conducted. Paraffin sections (5 μm) of eyes were collected and mounted on slides. Eyes were washed in xylene (2 × 10 min) and then rehydrated in 100% ethanol (2 × 5 min), 95% ethanol (1 × 5 min), 70% ethanol (3 min) and then water (5 min). For antigen retrieval, slides were heated for 5 min in a pre-heated 1× EDTA buffer (pH 8.5; Sigma–Aldrich, Sydney, Australia). After washing twice in PBS/0.1% Tween for 5 min, tissue sections were outlined with a paraffin pen. Sections were blocked with 3% H_2_O_2_ for 10 min, washed twice with PBS/0.1% Tween for 5 min and blocked with 5% FBS in PBS/0.1% Tween for 1 h in a humidified chamber. Sections were then incubated overnight at 4°C in a humidified chamber with goat anti-albumin antibody (1:200; Novus Biologicals, Colorado, U.S.A.) or goat anti-VEGF antibody (1:40; R&D Systems, Minneapolis, Canada) in PBS/0.1% Tween. Following overnight incubation, sections were washed three times with PBS/0.1% Tween for 5 min and incubated with rabbit anti-goat HRP (1:500, Thermo Fisher Scientific, Victoria, Australia) secondary antibodies conjugated with HRP in PBS/0.1% Tween for 1 h. This was followed by incubation with DAB. Slides were counterstained with Hematoxylin, dehydrated and mounted with DPX (Sigma–Aldrich, Sydney, Australia). Quantitation of albumin and VEGF staining was conducted using ImageJ 1.43j (National Institutes of Health, Bethesda, Maryland, U.S.A).

### Retinal RNA isolation and RNA sequencing

The total retinal RNA was extracted using TRIzol (Invitrogen, Massachusetts, U.S.A.) in accordance with the manufacturer’s protocol. TRIzol reagent-lysed retinas were mixed with chloroform and centrifuged to remove RNA upper aqueous phase from the lower organic phase. RNA in the upper aqueous phase was precipitated by isopropanol and centrifuged to precipitate RNA. This precipitated RNA was washed with 75% alcohol and dissolved in RNAse/DNAse-free water (Invitrogen). A Nanodrop ND-1000 spectrophotometer (Australian Biolab Group, Melbourne, Australia) was used to quantify the concentration of RNA. The RNA-Seq was performed by AGRF and the resulting RNA-Seq read was used to perform the analysis.

The Illumina Hi-seq 2500 platform was used for RNA-seq, and 100-bp reads were generated for the retinas. The data yield per individual mouse retina ranged from 2.18 to 3.56 Gb. The library for RNA-seq was prepared using Illumina’s Ribo Zero Gold protocol. The Illumina bcl2fastq 2.20.0.422 pipeline was used to generate primary read sequence with Illumina quality scores (phred-like quality + 33). The reads were screened for the presence of any Illumina adapter/overrepresented sequences and cross-species contamination. The reads were compared with the *Mus musculus* genome (Build version mm10) using the STAR aligner (v2.5.3a). The transcripts were assembled using the StringTie tool v2.1.4 using read alignment with M6 and reference annotation-based assembly option (RABT). Cufflinks, Cuffmerge and cuffdiff were employed for the purposes of differential expression analysis.

## Results

### Identification of SGLT2 in the eye of DR Akimba mice

**Figure 1:**
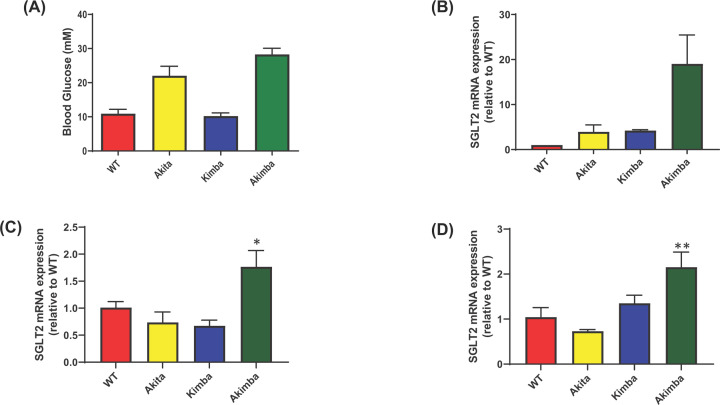
SGLT2 levels are elevated in the eyes of Akimba mice with DR (**A**) Blood glucose levels in mice displaying DR. (**B**) Identification of elevated *SGLT2* mRNA expression in the whole eyes of mice displaying DR. (**C**) Increased *SGLT2* mRNA expression in the kidney medulla of Akimba mice; *P=0.08 compared with WT. (**D**) Raised *SGLT2* mRNA expression in the kidney cortex of Akimba mice; **P=0.048 compared with WT. All data are expressed as mean + SEM; *n*=1–7 mice/group.

**Figure 2:**
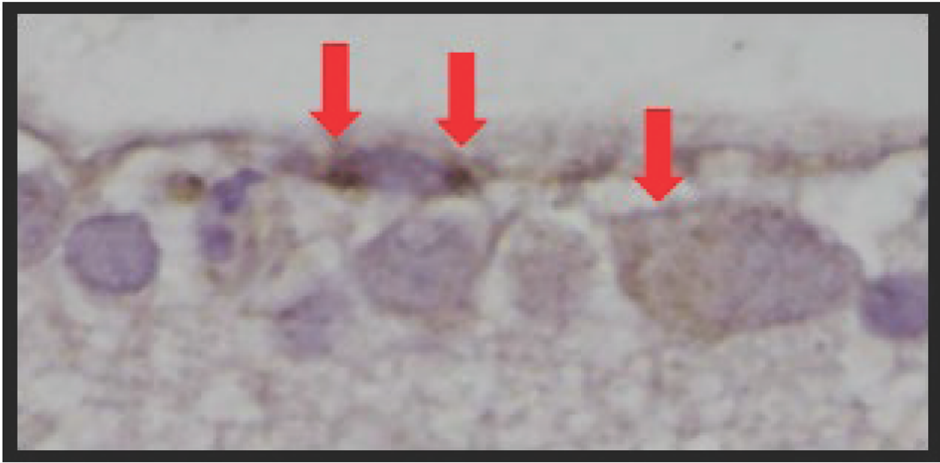
SGLT2 is expressed in the diabetic retina of vehicle-treated Akimba mice Positive SGLT2 staining (red arrows) in the retinal ganglion cell layer. Magnification 400×.

The Akimba mouse strain is an established DR mouse model. The strain is generated by crossing Kimba and Akita mice. The Akita and Akimba mice both display hyperglycemia (Figure 1A) but interestingly only the Akimba mice possess elevated levels of *SGLT2* mRNA in the eye and kidney (Figure 1B–D) highlighting the potential of SGLT2 as a valid therapeutic target for DR. Additionally, we were able to show that SGLT2 protein is expression in the retinal ganglion cells of the Akimba mice (Figure 2).

### EMPA treatment promotes glucose excretion in Kimba mice

Before treatment, urine glucose levels (via testing strips) were negative in the non-diabetic Kimba mice and positive in the diabetic Akimba mice. After 1 day of treatment, urine glucose levels tested positive in the Kimba mice receiving EMPA, while remaining negative in the Kimba mice receiving vehicle (Supplementary Figure S1). As expected, urine glucose levels of the diabetic Akimba mice were positive regardless of treatment.

### EMPA treatment was well-tolerated by mice

Kimba and Akimba mice displayed normal responses to touch, smooth and healthy body coats, bright eyes and normal posture throughout the 8 weeks of vehicle or EMPA treatment.

### EMPA treatment promoted weight gain and the ability to thrive in mice with DR

**Figure 3:**
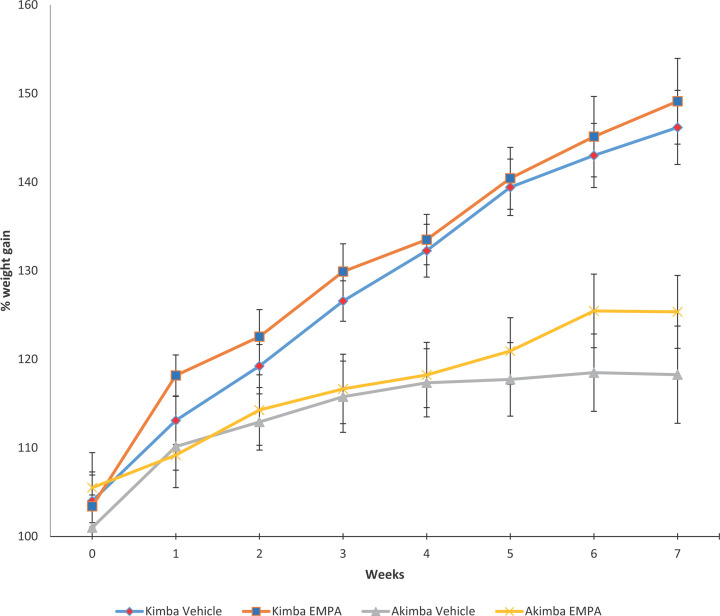
SGLT2 inhibition with EMPA promotes the ability to thrive in diabetic Akimba mice Normalized body weight percentages were calculated over 7 weeks of treatment. Mean ± SEM; *n*=6 mice/group.

Over the course of treatment, non-diabetic Kimba mice gained weight at similar rates irrespective of treatment (Figure 3). In diabetic Akimba mice, the SGLT2 inhibitor EMPA was shown to promote weight gain from weeks 5 to 7 (Figure 3).

### EMPA significantly reduced excessive water intake in diabetic Akimba mice

**Figure 4:**
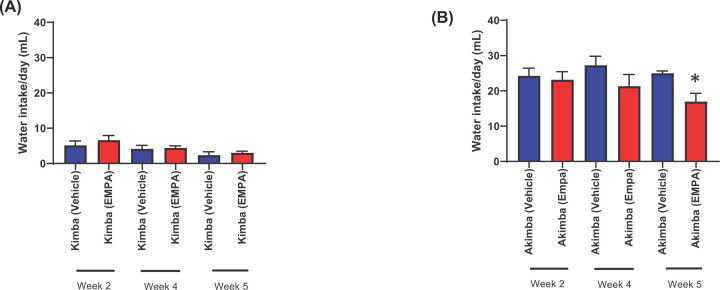
Inhibition of SGLT2 with EMPA significantly reduces excessive water consumption in diabetic Akimba mice Daily water intake of Kimba (**A**) and Akimba (**B**) mice was measured on weeks 2, 4 and 5 of treatment. *P=0.009 compared with week 5 vehicle. Mean ± SEM; *n*=6 mice/group; except *n*=4/group for week 5 Akimba data.

Throughout the duration of the experiment, the daily water intake for non-diabetic Kimba mice was lower than 10 ml/day (Figure 4A) and was markedly lower than the diabetic Akimba mice (Figure 4B). This confirms the non-diabetic status of the Kimba mice. There was also no difference in water intake between the Kimba (Vehicle) and Kimba (EMPA) groups (Figure 4A). After testing water levels at 2, 4 and 5 weeks post-treatment, the Akimba (EMPA) mice showed a decrease in water intake, in comparison to the Akimba (Vehicle) mice. At week 5, Akimba (EMPA) mice drank significantly less than the Akimba (Vehicle) mice, highlighting the improved diabetic phenotype (Figure 4B).

### EMPA reduces fasting blood glucose levels in diabetic mice

**Figure 5:**
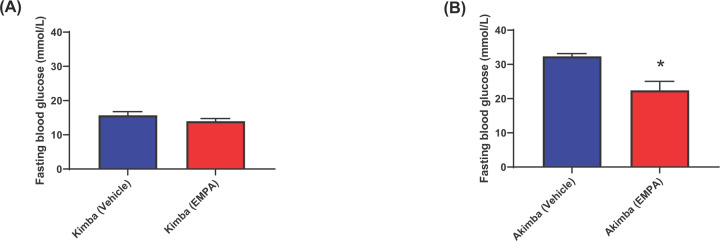
Fasting blood glucose levels are significantly reduced in diabetic Akimba mice after 8 weeks post-treatment Blood glucose levels in Kimba mice (**A**) and Akimba mice (**B**). *P=0.0037 compared with vehicle. Mean ± SEM; *n*=6 mice/group.

The Kimba blood glucose levels were consistently lower than 20 mmol/l (Figure 5A) and markedly lower than the diabetic Akimba mice (Figure 5B). This supports the non-diabetic status of the Kimba mice. In addition, there was no difference between the Kimba (Vehicle) and Kimba (EMPA) groups (Figure 5A). After 8 weeks of treatment, the fasting blood glucose levels of Akimba (EMPA) mice were significantly lower than the Akimba (Vehicle) mice (Figure 5B). This result highlights that EMPA was able to reduce diabetic hyperglycemia.

### The renal effects of EMPA in young diabetic mice

**Figure 6:**
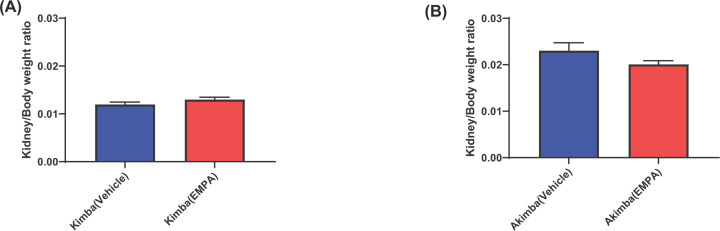
Kidney/body weight ratios are reduced in diabetic Akimba mice treated with EMPA Kimba mice (**A**) and Akimba mice (**B**). Mean ± SEM; *n*=6 mice/group.

The kidney-to-body weight ratios of Kimba mice (Figure 6A) were markedly lower than those in the Akimba mice and there was no effect of treatment in Kimba mice (Figure 6A). The lower kidney-to-body weight ratios confirmed the healthy renal phenotype of the Kimba mice. The increased kidney-to-body weight ratios in the Akimba mice confirmed the renal hypertrophy phenotype of the Akimba mouse. The Akimba (EMPA) mice displayed reduced kidney-to-body weight ratios compared with their vehicle-treated counterparts (Figure 6B), highlighting the renal benefits of EMPA.

### Ophthalmic benefits of SGLT2 inhibition in mice with DR

**Figure 7:**
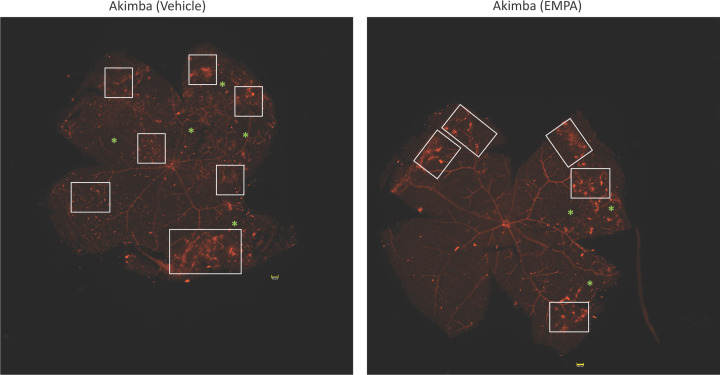
Retinal photomontages of Akimba mice treated with vehicle or EMPA Murine retinas were stained for vasculature. IRMAs are represented with boxes, and capillary dropouts are represented with asterisks. Magnification: 40×. Scale bar = 100 μm. Images are representative of *n*=6 mice/group.

**Figure 8:**
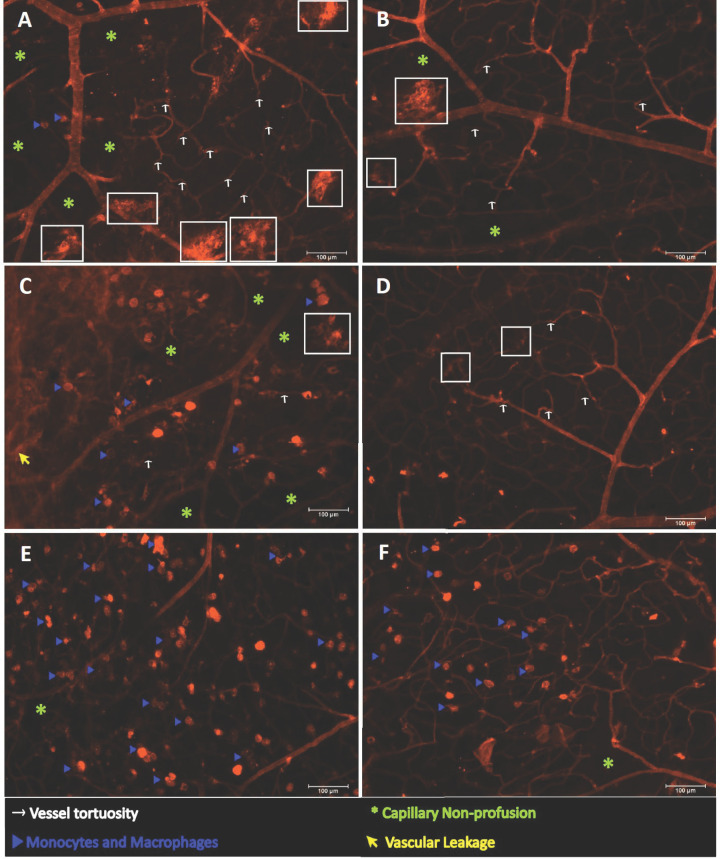
Representative areas of the retinal vasculature are shown in the upper, middle and lower capillary beds in young Akimba mice treated with Vehicle and EMPA Vehicle-treated retinas (**A**,**C**,**E**) showed extensively disorganized and disrupted vasculature. Vehicle-treated mice showed the presence of continuous and twisted capillaries that were folded on themselves and resembled large microaneurysms, vascular tufts and IRMAs (white boxes; A,C), tortuous vessels (white arrows; A,C) and capillary non-profusion (green asterisk; A,C,E). In addition, diffused staining indicative of loss of vessel integrity and leakage (yellow arrow; C) and wide distribution of monocytes/macrophages (blue arrow heads; A,C,E) were observed. EMPA-treated retinas (**B**,**D**,**F**) showed a better overall retinal vascular integrity and organization. The presence of smaller microaneurysms, vascular tufts and IRMAs (white boxes; B,D), less tortuous vessels (white arrows; B,D), minimal capillary non-profusion (green asterisk; B,F) and a lower distribution frequency of monocytes/macrophages (blue arrow heads; F) were observed. Scale bars = 100 μm.

As the aim of our study was to determine whether SGLT2 inhibition may prevent the development of DR, the severity of retinal vascular lesions was assessed 8 weeks post-treatment in Akimba mice treated with either vehicle or EMPA. The retinal vascular and pathological changes in EMPA and Vehicle-treated Akimba mice are compared and summarized in Table 1. Retinal whole mounts showing the vasculature of Akimba mice treated with vehicle compared with EMPA is illustrated in Figure 7. Table 1 was generated using measurements taken from similar images in Figures 7 and 8.

**Table 1:** Grading of retinal vascular lesions in young Akimba mice treated with Vehicle and EMPA

Characteristic	Akimba + Vehicle						Akimba + EMPA					
	1	2	3	4	5	6	1	2	3	4^7^	5	6
Microaneurysms/IRMA^1^	++^6^	++	++^6^	++	+	+	+	+	+	+	−/+	+
Capillary non-profusion^2^	++^6^	++	++^6^	++	++	++	+	+	+	−	++	++
Loss of vessel integrity and leakage^3^	+++	+	−	−	+	+	−	−	−	−	−	+
Vessel tortuosity and vessel tufts^4^	++^6^	++	++	++	+/++	++	+	+	+	−	−/+	+
Monocytes/macrophages^5^	++^6^	++	++^6^	+	−	++	+	+	+	−	+	++

1 Not present; +, less than 120 microaneurysms/IRMA present; ++, more than 120 microaneurysms/IRMA present.

2 No regions; +, less than eight regions; ++, more than eight regions.

3 Not present; +, mild; ++, moderate; +++, severe.

4 Absent; + moderate; ++, severe.

5 Absent; +, present in two or less retinal quadrant; ++, present in three or more retinal quadrant.

6 Expensive retinopathy/capillary dropout resulted in the retina showing lower incident of vascular lesions.

7 Showed less than 50 microaneurysms/IRMA and the overall retinal vasculature was clearly organized.

In Akimba mice treated with vehicle (Figure 7), the overall retinal vasculature was severely disrupted and disorganized. The retinas consistently showed the presence of torturous vessels (Figure 8A,C; white arrows). Vascular lesions such as microaneurysm, IRMA and neovascular tufts were large and were composed of continuous and contorted capillaries that were folded upon themselves (Figures 7 and 8A,C; white boxes) and were noted throughout the retina, without favoring any retinal quadrant or region. Both the upper and middle capillary beds showed increased capillary non-profusion (Figures 7 and 8A,C,E; green asterisks). Highlighting monocytes or macrophages, positivity was observed in small round structures where some had dendrites (Figure 8A,C,E; blue arrow heads). Diffused staining (Figure 8C; yellow arrow) in vehicle-treated Akimba mice indicated the loss of vessel integrity and leakage.

In contrast, we found that Akimba mice treated with EMPA showed less pronounced vascular disorganization (Figure 7) and showed a marked reduction in microaneurysms, IRMA, neovascular tufts (Figures 7 and 8B,D; white boxes) that was often limited to the peripheral retina. In addition, there was a reduction in vessel tortuosity (Figure 8B,D; white arrows), regions of capillary non-profusion (Figures 7 and 8B,F; green asterisks), monocytes or macrophages (Figure 8F; blue arrow heads) and vascular leakage was absent.

**Figure 9:**
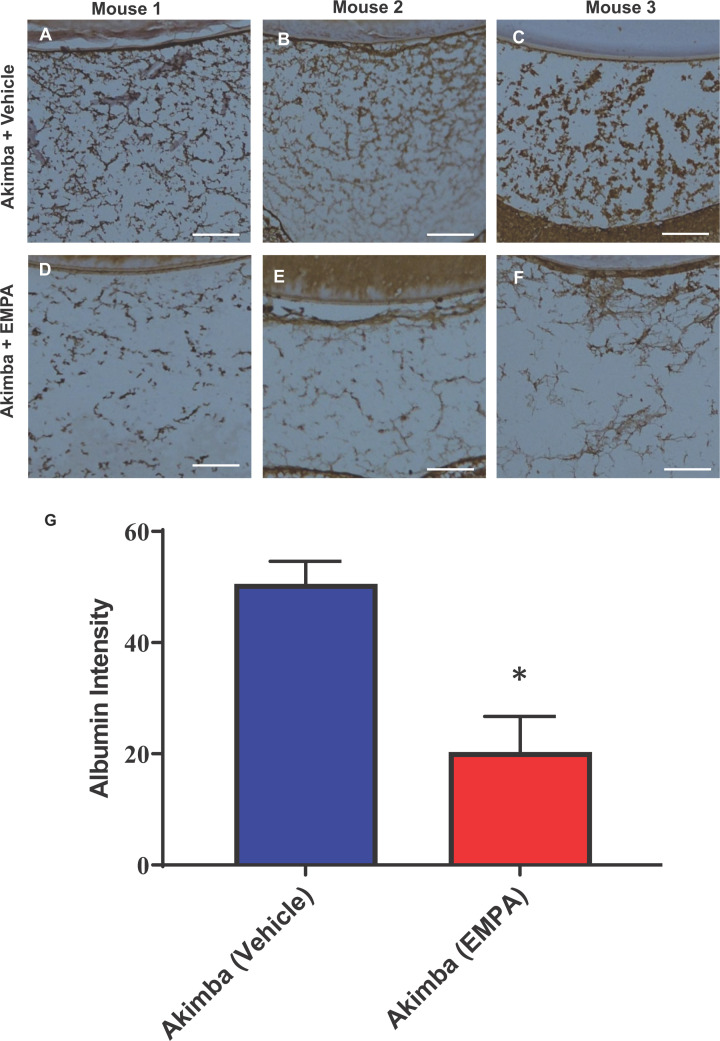
Assessment of vascular leakage in the eye Immunohistochemical staining for Albumin (indicated by brown staining) in the vitreous humor of diabetic Akimba mice treated with Vehicle (**A–C**) or EMPA (**D–F**). Representative images from 3 mice/group. Magnification 200×; scale bar = 100 μm. Quantitation of albumin in vitreous humor (**G**); *n*=4 mice/group; *P=0.0072.

We found that leakage of albumin was significantly decreased in vitreous humor in the EMPA-treated Akimba mice when compared with the vehicle-treated Akimba mice (Figure 9).

**Figure 10:**
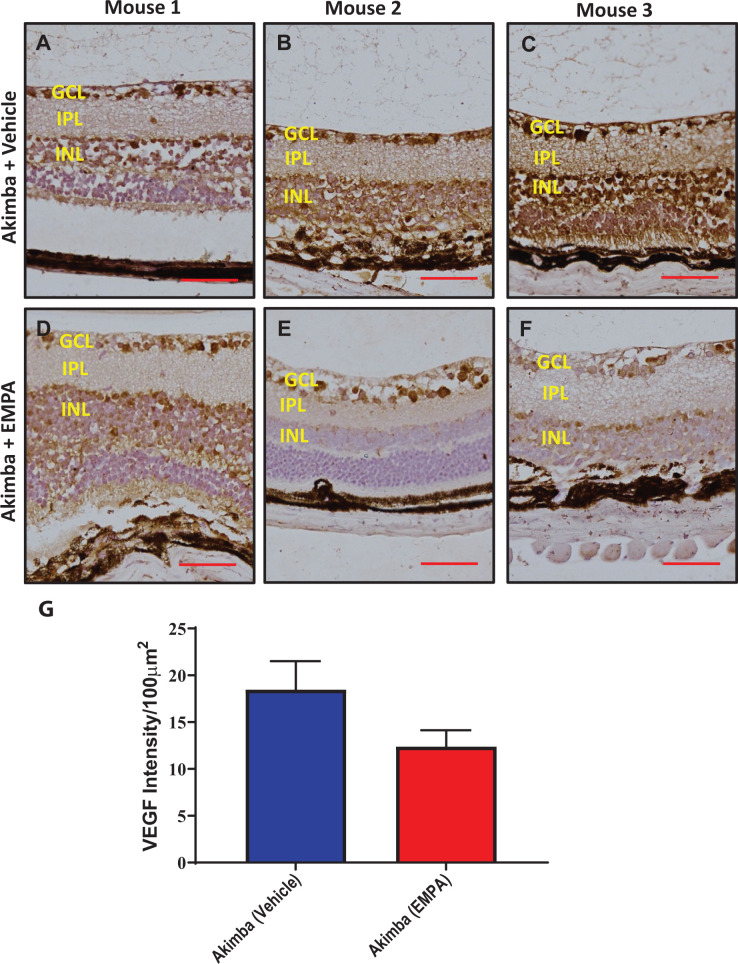
Detection of the angiogenic factor VEGF in the retina Immunohistochemical staining for VEGF (indicated by brown staining) in the retinas of diabetic Akimba mice treated with Vehicle (**A–C**) or EMPA (**D–F**). Representative images from 3 mice/group. Magnification 200×; scale bar = 100 μm. Quantitation of VEGF in retina (**G**); *n*=4 mice/group.

An intense VEGF expression was observed throughout the ganglion cell layer (GCL) of the retina. We observed a pronounced reduction in VEGF in the GCL, inner plexiform layer (IPL) and inner nuclear layer (INL) retinal layers of mice treated with EMPA (Figure 10).

In an effort to determine whether EMPA altered the retinal genetic signature, we performed RNA-Seq analysis. We found that there were many genes [27] that were both up- and down-regulated with SGLT2 inhibition (Supplementary Tables S1 and S2). Numerous targets were affected by SGLT2 inhibition. Some of these include *Tnfsf18*, *Ccr2* and *Il12rb1*. It is plausible that many of these genes may ultimately impact the pathogenesis of DR in our Akimba mouse model but requires further study.

### Mortality rates in Akimba mice

We also aimed to determine whether EMPA impacts mortality in the Akimba mouse model of DR. There was a 20% mortality rate (one out of five mice) in vehicle-treated Akimba mice, whereas 100% survival in Akimba mice treated with EMPA was noted. This result mimics the positive effects that SGLT2 inhibition with EMPA has on reducing mortality rates in human diabetics [20]. There was a 100% survival rate in all Kimba mice.

## Discussion

Diabetes is one of the fastest growing chronic metabolic diseases globally and the leading cause of preventable blindness. As such, therapies tackling underlying cardiovascular and renal complications and the related ophthalmic conditions like DR are urgently required. Although there is one study outlining the beneficial effects of the SGLT2 inhibitor, Ipragliflozin, on DR in male rats [21], there is overall limited data from either animal models or clinical trials regarding the effects of SGLT2 inhibition on DR. The aim in our current study was to explore whether SGLT2 inhibition in addition to its beneficial impact on glucose metabolism and insulin sensitivity, may also exert protective effects in regard to the development of DR.

We found that while the body weight of the non-diabetic Kimba mice was the same in both EMPA and Vehicle-treated groups, the diabetic Akimba mice exhibited an increase in weight between weeks 5 and 7 after EMPA treatment compared with vehicle-treated mice. One of the complications of type 1 diabetes (T1D) is a failure to thrive. Therefore, it is promising that EMPA prevents the weight loss associated with T1D. In fact, all mice exhibited normal behavior and showed no weight loss due to SGLT2 inhibition suggesting that SGLT2 inhibition does not promote detrimental side effects.

Highlighting the effectiveness of the SGLT2 inhibition therapy in our study, the fasting blood glucose levels in the diabetic Akimba mice treated with EMPA were significantly lower than the Akimba mice administered vehicle in their drinking water. Taking into account the glucose-lowering effects of EMPA along with the significant lowering of water intake in Akimba mice treated with EMPA, our findings highlight that EMPA reduces excessive thirst usually associated with diabetes and therefore shows improvement of the diabetic phenotype. However, SGLT2 inhibition did not completely halt diabetes development in Akimba mice. This is based on the observation that our Akimba mice which were obtained at a young age prior to demonstrating glucosuria (a natural hallmark of diabetic Akimba mice) subsequently all developed glucosuria over the course of our 8-week experimental regime.

Another complication associated with diabetes is renal failure. In the early stages of diabetes, the glomerular filtration rate (GFR) may be elevated and kidney size will be increased, due to heightened single-nephron GFR and expanded nephron size [22]. This enlargement in the kidney size may contribute to nephropathy and therefore renal failure. We have found in this study that the kidney-to-body weight ratio in the Akimba mice treated with EMPA was lower in comparison to the Akimba mice administered vehicle, therefore indicating the protective renal effects of EMPA in our young DR mouse model.

We demonstrated in our current study that Akimba mice also have elevated ocular expression of *SGLT2* compared with their parental Kimba and Akita mice (Figure 1). Therefore, we considered that SGLT2 inhibition may be a viable therapy for the prevention or reduction in the severity of DR. Excitingly, we showed that EMPA did improve the retinal vasculature in diabetic Akimba mice (Table 1; Figures 7 and 8). In order to further demonstrate the ocular benefits of EMPA, we assessed Albumin and VEGF retinal protein expression (Figures 9 and 10). Albumin is a marker of leakage [23] from the blood vessels whereby the albumin escapes from the blood vessels and binds to cells within the retina. VEGF is a marker which promotes blood vessel development and in DR there is an abundance of VEGF-mediated newly formed irregular blood vessels [24]. In our study, we demonstrated that EMPA reduces both Albumin and VEGF protein in the different layers of the retina to reduce the pathogenesis of DR. There is also a possibility that SGLT2 inhibition may occur in the kidney of Akimba mice and promote ocular benefits by cross-talk from the kidney. Hence, we show that there is potential for SGLT2 inhibitors to be used as a therapy to treat DR. Further studies are required to ascertain whether these promising findings in our specific DR mouse model are translatable to the human scenario and may indeed represent an effective therapeutic approach to halt the development of DR at an early stage of the disease process. A major therapeutic that is clinically used currently is intravitreal administration of anti-VEGF antibody. Utilizing this knowledge, it is plausible that SGLT2 inhibitors may also ultimately be administered intravitreally [25]. In DR, the blood–retinal barrier (BRB) is compromised and hyperglycemia may contribute to the BRB breakdown. Therefore, SGLT2 expression in the retina may be inhibited with SGLT2 inhibitors, reduce glucose reabsorption in the eye and ultimately decrease the damaging effects of hyperglycemia [26].

## Perspectives

- The present study investigated the potential utility of SGLT2 inhibition with EMPA to prevent or reduce the severity of early DR.
- When the SGLT2 inhibitor, EMPA, is given in the early stages of T1D, it may promote the ability to thrive by reducing the weight loss associated with T1D, significantly decreased diabetes-associated polydipsia (excessive thirst), significantly lowered fasting blood sugar levels, decreased kidney weights and most importantly, in the current context, reduced retinal abnormalities associated with early DR.
- We also show that EMPA reduces retinal vascular leakage indicated by lower albumin staining and diminishes expression of the pathogenic factor VEGF in the retina. Additionally, EMPA significantly alters the retinal genetic signature.
- As EMPA demonstrates beneficial outcomes in our young DR mouse model, it may be a potential therapeutic approach for patients with diabetes to prevent or reduce the severity of retinopathy, a proposition that now needs to be verified in adequately designed human studies.

## Supplementary Material

Supplementary Figure S1 and Tables S1-S2

## Data Availability

All supporting data are included within the main article and are available by contacting the corresponding author.

## Open Access

Open access for this article was enabled by the participation of The University of Western Australia in an all-inclusive *Read & Publish* agreement with Portland Press and the Biochemical Society under a transformative agreement with CAUL.

## Abbreviations

DAB: diaminobenzidine
DR: diabetic retinopathy
EMPA: Empagliflozin
GCL: ganglion cell layer
INS2: insulin 2
IRMA: intraretinal microvascular abnormality
PDR: proliferative DR
PFA: paraformaldehyde
SGLT2: sodium glucose transporter 2
T1D: type 1 diabetes
VEGF: vascular endothelial growth factor
