# Supplementary material for: Determining the role of SGLT2 inhibition with Empagliflozin in the development of diabetic retinopathy
Source: Biosci Rep. 2022 Mar 2;42(3):BSR20212209. doi: 10.1042/BSR20212209 (PMC8891593; doi:10.1042/BSR20212209)
Supplement: Supplementary Figure S1 and Tables S1-S2 [file BSR-2021-2209_supp.pdf]

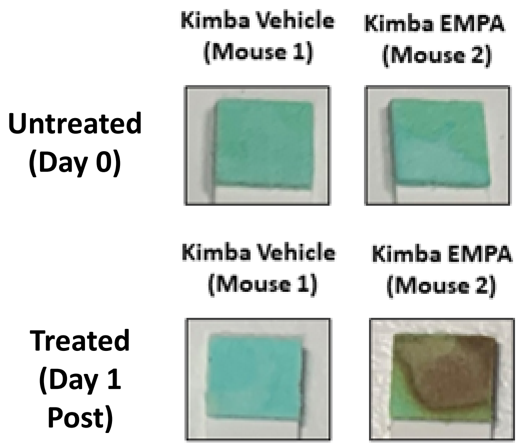

Supplementary Figure 1

Supplementary Figure 1

SGLT2 inhibition with EMPA promotes glucosuria in the non-diabetic Kimba mice. Urine glucose levels 1 day post treatment: blue (0 mM), brown ($\geq$ 111mM).

Gene symbol	Gene name	Function [27]	Fold change	P value
Cyp2a5	cytochrome P450, family 2, subfamily a 5	arachidonic acid epoxygenase activity	3.857240565	0.051503669
Kera	keratocan	cornea development in camera-type eye	3.742389644	0.019479387
Sln	sarcolipin	enzyme regulator activity	3.725250729	0.053350941
Cldn4	claudin 4	chloride channel activity, chloride transport	3.724254171	0.021719967
Siglecg	sialic acid binding Ig-like lectin G	adaptive immune response	3.666614651	0.01536648
Snora74a	small nucleolar RNA, H/ACA box 74A	biological process	3.66610682	0.016285591
Dnase2b	deoxyribonuclease II beta	deoxyribonuclease II activity	3.638864293	0.022619922
Tnfsf18	tumor necrosis factor (ligand) superfamily, member 18	immune response	3.631109631	0.026011525
Cldnd2	claudin domain containing 2	biological process	3.274848206	0.040599007
Csf3	colony stimulating factor 3 (granulocyte)	cytokine activity	3.266595584	0.044957601
Ccr2	chemokine (C-C motif) receptor 2	angiogenesis, cellular homeostasis	2.95884095	0.040011022
Rbp2	retinol binding protein 2, cellular	fatty acid binding and transport, lipid binding, retinal binding	2.343465742	0.045135505
Crygn	crystallin, gamma N	lens development in camera-type eye	1.090323443	0.033317793
Capn13	Calpain 13	calcium-dependent cysteine-type endopeptidase activity, proteolysis	3.349445394	0.040831222
Vmn2r2	vomeronasal 2, receptor 2	G protein-coupled receptor activity	3.321895849	0.042172927
Acsm1	acyl-CoA synthetase medium-chain family member 1	acyl-CoA metabolic process, ATP binding	3.31576368	0.040418542
Gucy2d	guanylate cyclase 2d	ATP binding, cGMP biosynthethic process	3.296558921	0.05027578
Cfhr2	complement factor H-related 2	regulation of complement activation	3.295712475	0.050133267
Mef2b	myocyte enhancer factor 2B	cell differentiation, transcription regulator complex	2.728570385	0.017852111
Cd200r3	CD200 receptor 3	regulation of neuroinflammatory response, protein binding	3.294094673	0.043557658
Slc26a9	solute carrier family 26, member 9	positive regulation of gene expression, ATPase binding	3.274467007	0.040783998
Tm4sf4	transmembrane 4 superfamily member 4	biological process	3.266058588	0.044985742
Cxcl11	chemokine (C-X-C motif) ligand 11	chemokine activity	2.698354012	0.026731546

Supplementary Table 1: Genes which are upregulated in Diabetic Akimba mice treated with EMPA.

List of up-regulated genes identified in gene expression analysis between EMPA and Vehicle treated Akimba mice. n=3 mice/group.

Gene symbol	Gene name	Function [27]	Fold change	P value
Il12rb1	interleukin 12 receptor, beta 1	cytokine binding, IL12R binding, positive regulation of activated T cell proliferation	-1.801369438	0.049798122
Gpr132	G protein-coupled receptor 132	signal transduction, negative regulation of G2/M transition of mitotic cell cycle	-2.478512667	0.045420965
Nkg7	natural killer cell group 7 sequence	biological process	-2.562122832	0.042873324
Hbq1a	hemoglobin, theta 1A	haptoglobin binding, oxygen binding	-3.553915532	0.04022278
Cd200r1	CD200 receptor 1	negative regulation of interleukin-6 production, macrophage and T cell migration	-3.881890464	0.043080023
Pck1	phosphoenolpyruvate carboxykinase 1, cytosolic	cellular response to insulin stimulus, gluconeogenesis	-3.920497257	0.029120686
Tfpi2	tissue factor pathway inhibitor 2	hemostasis	-4.764314802	0.001556838
Capn8	calpain 8	calcium-dependent cysteine-type endopeptidase activity	-3.151810943	0.052999586
Kap	kidney androgen regulated protein	extracellular region	-2.7618039	0.053316686

Supplementary Table 2: Genes which are downregulated in Diabetic Akimba mice treated with EMPA.

List of down-regulated genes identified in gene expression analysis between EMPA and Vehicle treated Akimba mice. n=3 mice/group.
